# Supplementary material for: The effect of listening to music on human transcriptome
Source: PeerJ. 2015 Mar 12;3:e830. doi: 10.7717/peerj.830 (PMC4362302; doi:10.7717/peerj.830)
Supplement: Figure S1 [file peerj-03-830-s001.pdf]

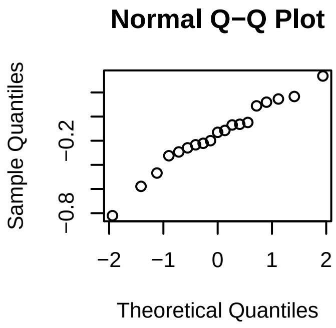
Normal Q−Q Plot
Sample Quantiles
−0.8
−0.2
Theoretical Quantiles
−2
−1
0
1
2

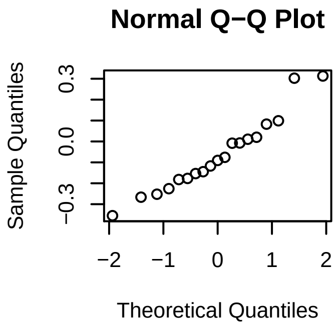
Normal Q−Q Plot
Sample Quantiles
−0.3
0.0
0.3
Theoretical Quantiles
−2
−1
0
1
2

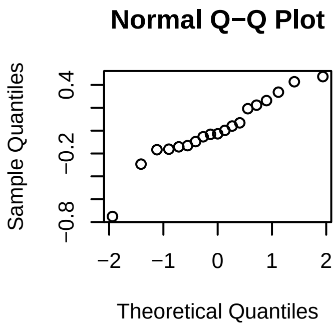
Normal Q−Q Plot
Sample Quantiles
−0.8
−0.2
0.4
Theoretical Quantiles
−2
−1
0
1
2

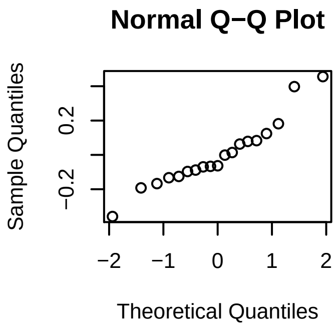
Normal Q−Q Plot
Sample Quantiles
−0.2
0.2
Theoretical Quantiles
−2
−1
0
1
2

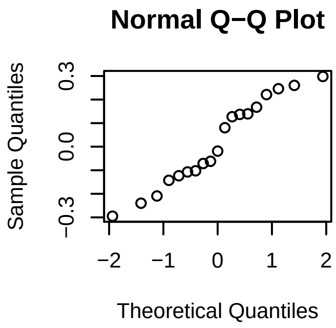
Normal Q−Q Plot
Sample Quantiles
−0.3
0.0
0.3
Theoretical Quantiles
−2
−1
0
1
2

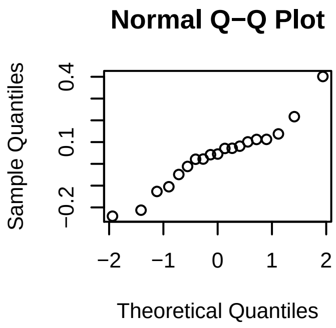
Normal Q−Q Plot
Sample Quantiles
−0.2
0.1
0.4
Theoretical Quantiles
−2
−1
0
1
2

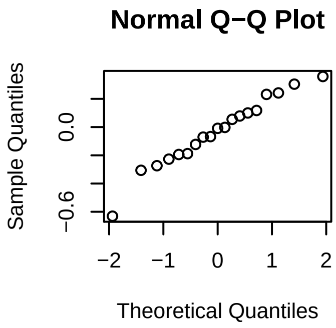
Normal Q−Q Plot
Sample Quantiles
−0.6
0.0
Theoretical Quantiles
−2
−1
0
1
2

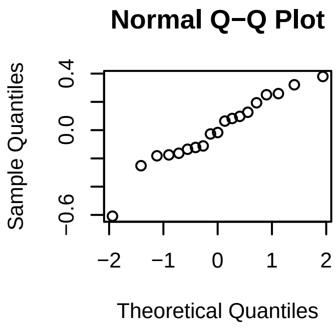
Normal Q−Q Plot
Sample Quantiles
−0.6
0.0
0.4
Theoretical Quantiles
−2
−1
0
1
2

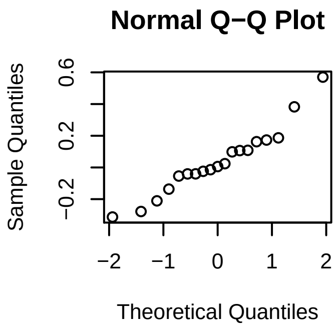
Normal Q−Q Plot
Sample Quantiles
−0.2
0.2
0.6
Theoretical Quantiles
−2
−1
0
1
2

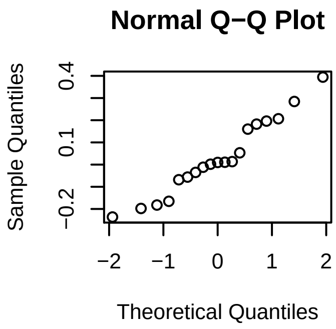
Normal Q−Q Plot
Sample Quantiles
−0.2
0.1
0.4
Theoretical Quantiles
−2
−1
0
1
2

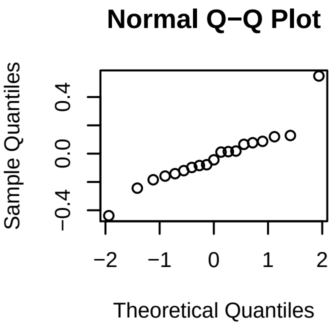
Normal Q−Q Plot
Sample Quantiles
0.4
0.0
−0.4
−2 −1 0 1 2
Theoretical Quantiles

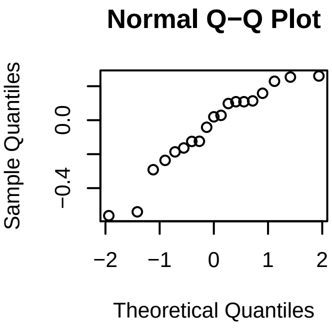
Normal Q−Q Plot
Sample Quantiles
0.0
−0.4
−2 −1 0 1 2
Theoretical Quantiles

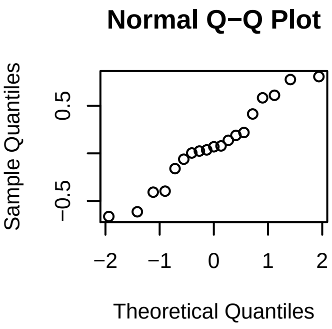
Normal Q−Q Plot
Sample Quantiles
0.5
−0.5
−2 −1 0 1 2
Theoretical Quantiles

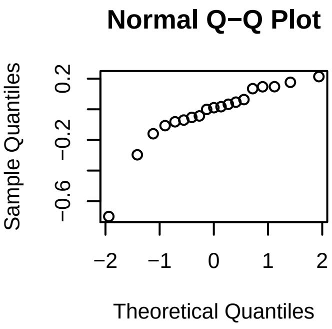
Normal Q−Q Plot
Sample Quantiles
0.2
−0.2
−0.6
−2 −1 0 1 2
Theoretical Quantiles

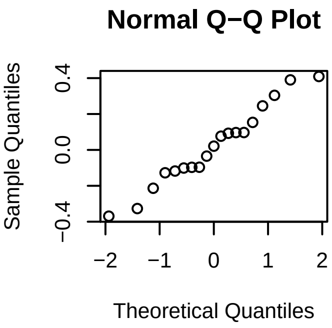
Normal Q−Q Plot
Sample Quantiles
0.4
0.0
−0.4
−2 −1 0 1 2
Theoretical Quantiles

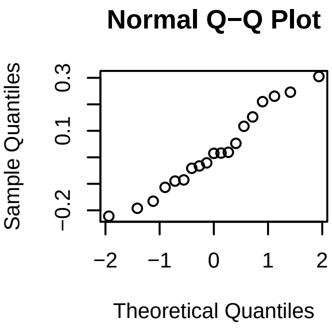
Normal Q−Q Plot
Sample Quantiles
0.3
0.1
−0.2
−2 −1 0 1 2
Theoretical Quantiles

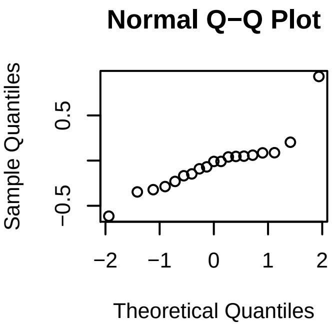
Normal Q−Q Plot
Sample Quantiles
0.5
−0.5
−2 −1 0 1 2
Theoretical Quantiles

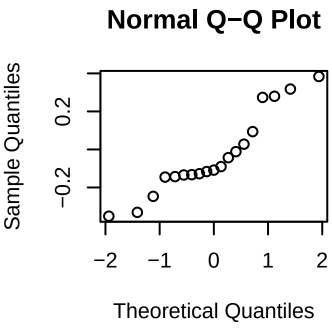
Normal Q−Q Plot
Sample Quantiles
0.2
−0.2
−2 −1 0 1 2
Theoretical Quantiles

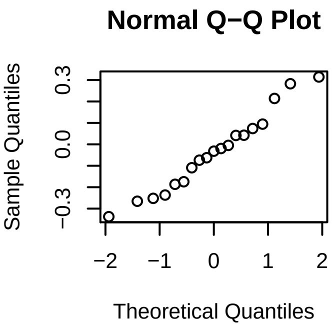
Normal Q−Q Plot
Sample Quantiles
0.3
0.0
−0.3
−2 −1 0 1 2
Theoretical Quantiles

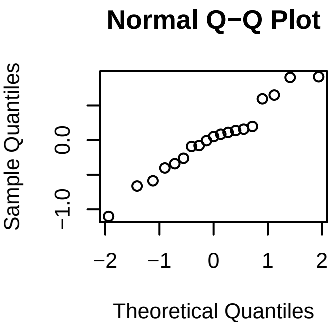
Normal Q−Q Plot
Sample Quantiles
0.0
−1.0
−2 −1 0 1 2
Theoretical Quantiles

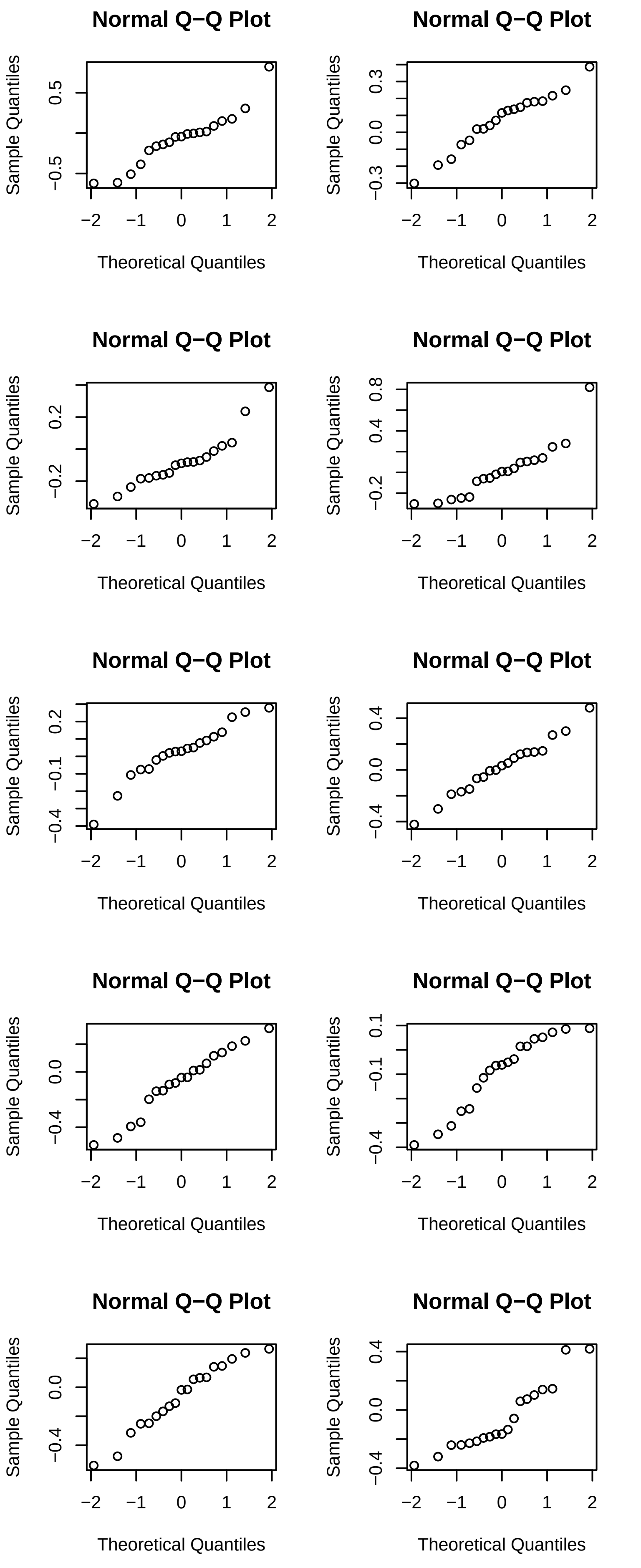

Normal Q−Q Plot
Sample Quantiles
−0.5
0.5
−2
−1
0
1
2
Theoretical Quantiles
Normal Q−Q Plot
Sample Quantiles
−0.3
0.0
0.3
−2
−1
0
1
2
Theoretical Quantiles
Normal Q−Q Plot
Sample Quantiles
−0.2
0.2
−2
−1
0
1
2
Theoretical Quantiles
Normal Q−Q Plot
Sample Quantiles
−0.2
0.4
0.8
−2
−1
0
1
2
Theoretical Quantiles
Normal Q−Q Plot
Sample Quantiles
−0.4
−0.1
0.2
−2
−1
0
1
2
Theoretical Quantiles
Normal Q−Q Plot
Sample Quantiles
−0.4
0.0
0.4
−2
−1
0
1
2
Theoretical Quantiles
Normal Q−Q Plot
Sample Quantiles
−0.4
0.0
−2
−1
0
1
2
Theoretical Quantiles
Normal Q−Q Plot
Sample Quantiles
−0.4
−0.1
0.1
−2
−1
0
1
2
Theoretical Quantiles
Normal Q−Q Plot
Sample Quantiles
−0.4
0.0
−2
−1
0
1
2
Theoretical Quantiles
Normal Q−Q Plot
Sample Quantiles
−0.4
0.0
0.4
−2
−1
0
1
2
Theoretical Quantiles

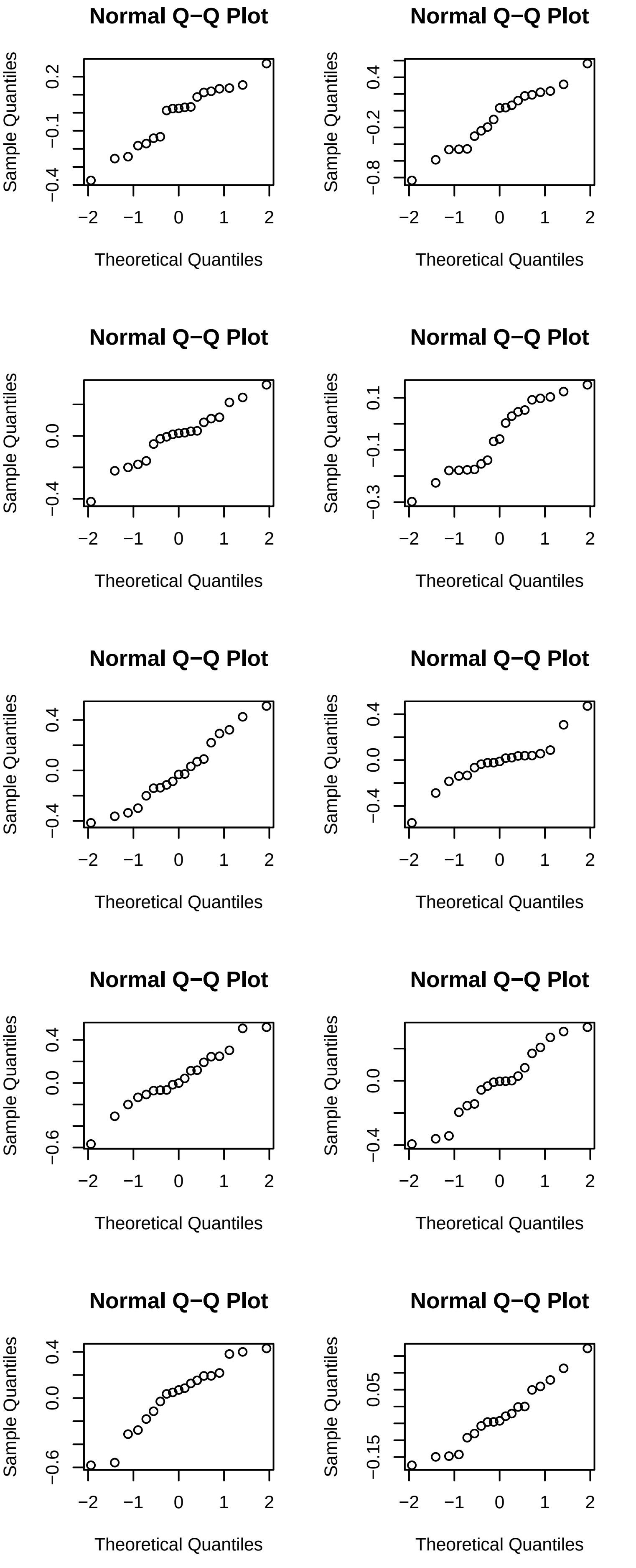
Normal Q−Q Plot
Sample Quantiles
Theoretical Quantiles
