# Supplementary material for: The effect of listening to music on human transcriptome
Source: PeerJ. 2015 Mar 12;3:e830. doi: 10.7717/peerj.830 (PMC4362302; doi:10.7717/peerj.830)
Supplement: Table S4 [file peerj-03-830-s005.docx]

| **Gene** | **Known Biological function** |
| --- | --- |
| *SNCA* | Involved in dopamine (DA) neuronal homeostasis (1, 2); Regulates the maintenance of mature synapses, and the stabilization of synaptic function (2). Essential for synaptic plasticity via the genesis and maintenance of presynaptic vesicles (3). The normal function of *SNCA* is regulated by neural activity and the response of *SNCA* depends upon the stimulus intensity (4). Described as the protein that is regulated during a critical period of song learning in songbirds (5); Located on chromosome 4q22.1 that shows significant linkage to musical aptitude (6, 7); Furthermore, *GATA2,* which is located in the most significant region of association with musical aptitude at 3q21 (6), is abundantly expressed in dopaminergic neurons and binds to intron-1 of endogenous neuronal *SNCA* to regulate its expression (8); Reduces oxidative stress and has a neuroprotective role by weakening the dopamine transporter activity (9) |
| *NPTN* | The *neuroplastin* (*NPTN*) gene encodes a cell adhesion protein that is known to regulate neuritogenesis and synaptic plasticity (10). Recent evidence suggests that *NPTN* was expressed in the brain during periods of intense neuronal activation and synaptic activity, and adolescents with highly functioning *NPTN* performed better in intelligence tests (11). Emerging evidence also suggests that *NPTN*, along with a few other genes, is a direct target of the *FOXP2* gene (12). |
| *FKBP8* | *FKBP8* may have a functional role in neuronal memory (Entrez: 23770). *FKBP8* is known to interact with lysosomal protein *ATP13A2* (*PARK9*), which prevents the misfolding and toxicity of *SNCA* (13). Moreover, *FKBP8* has also been suggested to exhibit neurotrophic and neuroprotective functions through the inhibition of apoptosis (14). Involved in vesicular trafficking, and interacts with SNARE complex. |
| *UBE2B* | Found to be regulated during song learning and singing in songbirds (15). |
| *LOC644936* | Beta-actin pseudogene. Beta-actin has been implicated in song learning and singing (16). Beta-actin is also crucial in auditory hair cell development and function (17, 18). Genes related to auditory hair cells have been associated with musical aptitude (6). |
| *NRGN* | Neurogranin has been described as the molecule expressed in the adult and developing song control system of songbirds (19). Furthermore *NRGN* also enhances LTP through calcium-mediated signaling (20), and *NRGN*-knockout mice exhibit significant performance deficits in learning (21). Increases synaptic strength, and regulates hippocampal synaptic plasticity and function. |
| *RTN4* | Also known as Nogo, this gene has been shown to modulate dopaminergic and motor functions (22) and alter spatial memory and social interaction (23). *RTN4* is also known to interact with *BACE1* and inhibit its ability to generate amyloid beta peptide and subsequently amyloid plaques, which are characteristic of neurodegenerative diseases such as Alzheimer's (24). |
| *LRRFIP1* | A transcription regulator that has been shown to be located in the strongest linkage region of human auditory cortical activation (25). |
| *AHAS2* | A co-chaperone that stimulates *HSP90* ATPase activity (Entrez: 130872) demonstrated in song learning and singing (15). |
| *PPP1R12A* | Functional and/or structural plasticity related gene; the expression is altered by pleasure in amygdala (26). |
| *RGS2* | This immediate early gene has been implicated in song learning and singing in songbirds (27) and has been shown to regulate emotions (28). This gene is rapidly induced in the hippocampus, cortex and striatum in response to stimuli that evokes plasticity (29). Plays a key role in regulating the intracellular signaling of G-protein coupled receptors such as dopamine and serotonin (30). |
| *FAM49B* | Located on chromosome 8q24.1 that shows the strongest linkage to absolute pitch (31) and has been demonstrated to be associated with musical aptitude in a genome-wide CNV scan (32). |
| *HDAC4* | A histone deacetylase located on chromosome 2q37.3, in the strongest linkage region of human auditory cortical activation (25). HDAC is also known to act as an epigenetic brake in critical period of learning. Modulation of *HDAC4* in acquiring absolute pitch has been demonstrated (33). *HDAC4* has also been described as the central molecule that governs a transcriptional program that is essential for experience-dependent synaptic plasticity and memory (34). Changes in the activities of histone family of proteins have been shown to shape the transcriptional responses to neuronal activity. Histone modifications have been implictaed in stimulus-dependent regulation of transcription. (35). |
| *KLF4* | A zinc-finger containing transcription factor, an immediate early response gene that regulates neurite outgrowth, axon growth and neuronal survival (36) and has been shown to be activated after glutamatergic synaptic activation (37, 38). Zinc-finger containing immediate early response genes have been implicated in song learning and singing in songbirds (16, 39–42). |
| *FTH1P8* | A pseudogene for ferritin polypeptide; Importantly, ferritin is regulated by *FOXP2*, a gene that has been implicated in song learning and singing in songbirds (43, 44). *FTH1* gene is involved ion channel activity and plays a crucial role in iron homeostasis within the brain (45). Defects in ferritin proteins are associated with several neurodegenerative diseases (45). Up-regulation of iron-regulating genes has been correlated with cognitive functions (46). |
| *MYC* | This immediate early response gene and transcription regulator has also been described in song control nuclei of songbirds (47). |
| *SLC6A8* | This creatinine transporter gene acts as a key synaptic regulator and neuroprotective agent that is important for neuronal function. *SLC6A8* deficiency is known to cause speech and language delay and X-linked intellectual disability (48–51). |
| *RAPGEF2* | Functions as a guanine nucleotide exchange factor, which activates Rap and Ras family of GTPases in a cAMP-dependent mannaer. A gene from the same gene family, with similar function, *ARHGEF9*, has been found to be regulated during singing in songbird (16). |
| *GRIPAP1* | Functions as a guanine nucleotide exchange factor for Ras family of GTPases that interacts with glutamate receptor interacting protein (*GRIP1*) and regulates the *AMPA* receptor function. A gene with similar function, *ARHGEF9*, has been found to be regulated during singing in songbird (16). |
| *TBC1D9* | Activates Rab family of GTPases. A gene with similar function, *ARHGEF9*, has been found to be regulated during singing in songbird (16). |
| *RALGAPA1* | Acts as a GTPase activator for Ras-like small GTPases. A gene with similar function, *ARHGEF9*, has been found to be regulated during singing in songbird (16). Also this gene, located on 14q13 known to cause developmental delay (52, 53). |
| *JMJD1C* | A histone demethylase that also acts as hormone-dependent transcription regulator (54, 55). Histone modifications play a key role in stimulus-dependent regulation of transcription (35), and changes in chromatin structure and histone family proteins have been shown to be regulated in song learning and singing in songbirds (15). |
| *APOBEC3A* | A gene that plays a crucial role in active DNA demethylation pathway and such epigenetic modifications are known to alter the gene transcription, leading to various neuropsychiatric diseases. For example, recent evidence shows that the expression of *APOBEC3A* has been decreased drastically in the parietal cortex of psychosis patients (56). |
| *ZNF83* | A C_2_H_2_ type zinc finger protein with no known function. Interestingly, another C_2_H_2_ type zinc finger protein, *ZNF225* (*ZENK*; *EGR1*) has been repeatedly described as the immediate-early response gene in songbirds during song learning and singing. Along with *FOS*, *ZENK* is the most well-documented gene in songbirds (16, 39–42). |
| *ZNF91* | A C_2_H_2_ type zinc finger protein with no known function. Interestingly, another C_2_H_2_ type zinc finger protein, *ZNF225* (*ZENK*; *EGR1*) has been repeatedly described as the immediate-early response gene in songbirds during song learning and singing. Along with *FOS*, *ZENK* is the most well-documented gene in songbirds (16, 39–42). |
| *ZNF483* | A C_2_H_2_ type zinc finger protein with no known function. Interestingly, another C_2_H_2_ type zinc finger protein, *ZNF225* (*ZENK*; *EGR1*) has been repeatedly described as the immediate-early response gene in songbirds during song learning and singing. Along with *FOS*, *ZENK* is the most well-documented gene in songbirds (16, 39–42). |
| *SEP15* | Selenoproteins execute the functions of essential micronutrient selenium, and deficiency of selenoproteins results in altered hippocampal synaptic function and sensorimotor gating (57, 58). |
| *DUSP6* | Located on 12q22-q23, an important candidate region for Schizophrenia and Bipolar disorder (59). |
| *SMNDC1* | Encodes a protein that contains domain for the ‘survival of motor neuron proetin’, which promotes axonal growth and extension (60, 61). |
| *NEDD9* | This gene has been shown to influence the cognitive function and risk for Alzheimer’s disease (62). |
| *S100A12* | A calcium binding protein is known to be involved in neurite outgrowth and neurogenesis (63). |

**References**

1. Oczkowska A, Kozubski W, Lianeri M, Dorszewska J (2013) Mutations in PRKN and SNCA Genes Important for the Progress of Parkinson’s Disease. *Curr Genomics* 14:502–17. Available at: http://www.ncbi.nlm.nih.gov/pubmed/24532983 [Accessed April 24, 2014].

2. Murphy DD, Rueter SM, Trojanowski JQ, Lee VM (2000) Synucleins are developmentally expressed, and alpha-synuclein regulates the size of the presynaptic vesicular pool in primary hippocampal neurons. *J Neurosci* 20:3214–20. Available at: http://www.ncbi.nlm.nih.gov/pubmed/10777786 [Accessed April 8, 2014].

3. Abeliovich A et al. (2000) Mice lacking alpha-synuclein display functional deficits in the nigrostriatal dopamine system. *Neuron* 25:239–52. Available at: http://www.ncbi.nlm.nih.gov/pubmed/10707987 [Accessed April 8, 2014].

4. Fortin DL et al. (2005) Neural activity controls the synaptic accumulation of alpha-synuclein. *J Neurosci* 25:10913–21. Available at: http://www.ncbi.nlm.nih.gov/pubmed/16306404 [Accessed April 2, 2014].

5. George JM, Jin H, Woods WS, Clayton DF (1995) Characterization of a novel protein regulated during the critical period for song learning in the zebra finch. *Neuron* 15:361–72. Available at: http://www.ncbi.nlm.nih.gov/pubmed/7646890 [Accessed April 3, 2014].

6. Oikkonen J et al. (2014) A genome-wide linkage and association study of musical aptitude identifies loci containing genes related to inner ear development and neurocognitive functions. *Mol Psychiatry*. Available at: http://www.ncbi.nlm.nih.gov/pubmed/24614497 [Accessed March 19, 2014].

7. Pulli K et al. (2008) Genome-wide linkage scan for loci of musical aptitude in Finnish families: evidence for a major locus at 4q22. *J Med Genet* 45:451–456. Available at: http://www.ncbi.nlm.nih.gov/pubmed/18424507.

8. Scherzer CR et al. (2008) GATA transcription factors directly regulate the Parkinson’s disease-linked gene alpha-synuclein. *Proc Natl Acad Sci U S A* 105:10907–12. Available at: http://www.pubmedcentral.nih.gov/articlerender.fcgi?artid=2504800&tool=pmcentrez&rendertype=abstract [Accessed April 24, 2014].

9. Wersinger C, Sidhu A (2003) Attenuation of dopamine transporter activity by alpha-synuclein. *Neurosci Lett* 340:189–92. Available at: http://www.ncbi.nlm.nih.gov/pubmed/12672538 [Accessed April 8, 2014].

10. Smalla KH et al. (2000) The synaptic glycoprotein neuroplastin is involved in long-term potentiation at hippocampal CA1 synapses. *Proc Natl Acad Sci U S A* 97:4327–32. Available at: http://www.pubmedcentral.nih.gov/articlerender.fcgi?artid=18241&tool=pmcentrez&rendertype=abstract [Accessed April 8, 2014].

11. Desrivières S et al. (2014) Single nucleotide polymorphism in the neuroplastin locus associates with cortical thickness and intellectual ability in adolescents. *Mol Psychiatry*. Available at: http://www.ncbi.nlm.nih.gov/pubmed/24514566 [Accessed April 8, 2014].

12. Vernes SC et al. (2011) Foxp2 regulates gene networks implicated in neurite outgrowth in the developing brain. *PLoS Genet* 7:e1002145. Available at: http://www.pubmedcentral.nih.gov/articlerender.fcgi?artid=3131290&tool=pmcentrez&rendertype=abstract [Accessed March 25, 2014].

13. Usenovic M et al. (2012) Identification of novel ATP13A2 interactors and their role in α-synuclein misfolding and toxicity. *Hum Mol Genet* 21:3785–94. Available at: http://www.pubmedcentral.nih.gov/articlerender.fcgi?artid=3412378&tool=pmcentrez&rendertype=abstract [Accessed March 30, 2014].

14. Shirane M, Nakayama KI (2003) Inherent calcineurin inhibitor FKBP38 targets Bcl-2 to mitochondria and inhibits apoptosis. *Nat Cell Biol* 5:28–37.

15. Wada K et al. (2006) A molecular neuroethological approach for identifying and characterizing a cascade of behaviorally regulated genes. *Proc Natl Acad Sci U S A* 103:15212–7. Available at: http://www.pubmedcentral.nih.gov/articlerender.fcgi?artid=1622802&tool=pmcentrez&rendertype=abstract [Accessed June 12, 2014].

16. Wada K et al. (2006) A molecular neuroethological approach for identifying and characterizing a cascade of behaviorally regulated genes. *Proc Natl Acad Sci U S A* 103:15212–7. Available at: http://www.pnas.org/content/103/41/15212.full [Accessed June 12, 2014].

17. Perrin BJ, Sonnemann KJ, Ervasti JM (2010) β-actin and γ-actin are each dispensable for auditory hair cell development but required for Stereocilia maintenance. *PLoS Genet* 6:e1001158. Available at: http://dx.plos.org/10.1371/journal.pgen.1001158 [Accessed May 27, 2014].

18. Perrin BJ et al. (2013) β-Actin and fascin-2 cooperate to maintain stereocilia length. *J Neurosci* 33:8114–21. Available at: http://www.pubmedcentral.nih.gov/articlerender.fcgi?artid=3718021&tool=pmcentrez&rendertype=abstract [Accessed May 27, 2014].

19. Clayton DF, George JM, Mello C V, Siepka SM Conservation and expression of IQ-domain-containing calpacitin gene products (neuromodulin/GAP-43, neurogranin/RC3) in the adult and developing oscine song control system. *Dev Neurobiol* 69:124–40. Available at: http://www.ncbi.nlm.nih.gov/pubmed/19023859 [Accessed July 7, 2014].

20. Huang K-P et al. (2004) Neurogranin/RC3 enhances long-term potentiation and learning by promoting calcium-mediated signaling. *J Neurosci* 24:10660–9. Available at: http://www.ncbi.nlm.nih.gov/pubmed/15564582 [Accessed March 21, 2014].

21. Pak JH et al. (2000) Involvement of neurogranin in the modulation of calcium/calmodulin-dependent protein kinase II, synaptic plasticity, and spatial learning: a study with knockout mice. *Proc Natl Acad Sci U S A* 97:11232–7. Available at: http://www.pubmedcentral.nih.gov/articlerender.fcgi?artid=17183&tool=pmcentrez&rendertype=abstract [Accessed April 2, 2014].

22. Willi R, Aloy EM, Yee BK, Feldon J, Schwab ME (2009) Behavioral characterization of mice lacking the neurite outgrowth inhibitor Nogo-A. *Genes Brain Behav* 8:181–92. Available at: http://www.ncbi.nlm.nih.gov/pubmed/19077178 [Accessed June 5, 2014].

23. Lazar NL et al. (2011) Missense mutation of the reticulon-4 receptor alters spatial memory and social interaction in mice. *Behav Brain Res* 224:73–9. Available at: http://www.ncbi.nlm.nih.gov/pubmed/21645550 [Accessed July 7, 2014].

24. He W et al. (2004) Reticulon family members modulate BACE1 activity and amyloid-beta peptide generation. *Nat Med* 10:959–965.

25. Renvall H et al. (2012) Genome-wide linkage analysis of human auditory cortical activation suggests distinct loci on chromosomes 2, 3, and 8. *J Neurosci* 32:14511–8. Available at: http://www.ncbi.nlm.nih.gov/pubmed/23077036 [Accessed July 8, 2014].

26. Ulrich-Lai YM et al. (2010) Pleasurable behaviors reduce stress via brain reward pathways. *Proc Natl Acad Sci U S A* 107:20529–34. Available at: http://www.pubmedcentral.nih.gov/articlerender.fcgi?artid=2996660&tool=pmcentrez&rendertype=abstract [Accessed May 23, 2014].

27. Clayton DF (2000) The genomic action potential. *Neurobiol Learn Mem* 74:185–216. Available at: http://www.ncbi.nlm.nih.gov/pubmed/11031127 [Accessed May 29, 2014].

28. Bendesky A, Bargmann CI (2011) Genetic contributions to behavioural diversity at the gene-environment interface. *Nat Rev Genet* 12:809–20. Available at: http://www.ncbi.nlm.nih.gov/pubmed/22064512 [Accessed May 25, 2014].

29. Han J et al. (2006) RGS2 determines short-term synaptic plasticity in hippocampal neurons by regulating Gi/o-mediated inhibition of presynaptic Ca2+ channels. *Neuron* 51:575–86. Available at: http://www.ncbi.nlm.nih.gov/pubmed/16950156 [Accessed July 8, 2014].

30. Ingi T et al. (1998) Dynamic regulation of RGS2 suggests a novel mechanism in G-protein signaling and neuronal plasticity. *J Neurosci* 18:7178–88. Available at: http://www.ncbi.nlm.nih.gov/pubmed/9736641 [Accessed June 4, 2014].

31. Theusch E, Basu A, Gitschier J (2009) Genome-wide study of families with absolute pitch reveals linkage to 8q24.21 and locus heterogeneity. *Am J Hum Genet* 85:112–9. Available at: http://www.pubmedcentral.nih.gov/articlerender.fcgi?artid=2706961&tool=pmcentrez&rendertype=abstract [Accessed July 8, 2014].

32. Ukkola-Vuoti L et al. (2013) Genome-Wide Copy Number Variation Analysis in Extended Families and Unrelated Individuals Characterized for Musical Aptitude and Creativity in Music. *PLoS One* 8:e56356. Available at: http://www.ncbi.nlm.nih.gov/pubmed/23460800.

33. Gervain J et al. (2013) Valproate reopens critical-period learning of absolute pitch. *Front Syst Neurosci* 7:102. Available at: http://www.pubmedcentral.nih.gov/articlerender.fcgi?artid=3848041&tool=pmcentrez&rendertype=abstract [Accessed May 28, 2014].

34. Sando R et al. (2012) HDAC4 governs a transcriptional program essential for synaptic plasticity and memory. *Cell* 151:821–34. Available at: http://www.pubmedcentral.nih.gov/articlerender.fcgi?artid=3496186&tool=pmcentrez&rendertype=abstract [Accessed June 10, 2014].

35. West AE, Greenberg ME (2011) Neuronal activity-regulated gene transcription in synapse development and cognitive function. *Cold Spring Harb Perspect Biol* 3. Available at: http://www.pubmedcentral.nih.gov/articlerender.fcgi?artid=3098681&tool=pmcentrez&rendertype=abstract [Accessed June 7, 2014].

36. Moore DL et al. (2009) KLF family members regulate intrinsic axon regeneration ability. *Science* 326:298–301. Available at: http://www.pubmedcentral.nih.gov/articlerender.fcgi?artid=2882032&tool=pmcentrez&rendertype=abstract [Accessed May 30, 2014].

37. Zhu S, Tai C, MacVicar BA, Jia W, Cynader MS (2009) Glutamatergic stimulation triggers rapid Krüpple-like factor 4 expression in neurons and the overexpression of KLF4 sensitizes neurons to NMDA-induced caspase-3 activity. *Brain Res* 1250:49–62. Available at: http://www.ncbi.nlm.nih.gov/pubmed/19041854 [Accessed July 8, 2014].

38. Zhang S-J et al. (2007) Decoding NMDA receptor signaling: identification of genomic programs specifying neuronal survival and death. *Neuron* 53:549–62. Available at: http://www.ncbi.nlm.nih.gov/pubmed/17296556 [Accessed May 28, 2014].

39. Mello C V, Clayton DF (1994) Song-induced ZENK gene expression in auditory pathways of songbird brain and its relation to the song control system. *J Neurosci* 14:6652–66. Available at: http://www.ncbi.nlm.nih.gov/pubmed/7965067 [Accessed July 4, 2014].

40. Mello C V, Ribeiro S (1998) ZENK protein regulation by song in the brain of songbirds. *J Comp Neurol* 393:426–38. Available at: http://www.ncbi.nlm.nih.gov/pubmed/9550149 [Accessed July 4, 2014].

41. Thode C, Bock J, Braun K, Darlison MG (2005) The chicken immediate-early gene ZENK is expressed in the medio-rostral neostriatum/hyperstriatum ventrale, a brain region involved in acoustic imprinting, and is up-regulated after exposure to an auditory stimulus. *Neuroscience* 130:611–7. Available at: http://www.sciencedirect.com/science/article/pii/S0306452204009558 [Accessed July 4, 2014].

42. Jarvis ED, Nottebohm F (1997) Motor-driven gene expression. *Proc Natl Acad Sci U S A* 94:4097–102. Available at: http://www.pubmedcentral.nih.gov/articlerender.fcgi?artid=20574&tool=pmcentrez&rendertype=abstract [Accessed July 4, 2014].

43. Vernes SC et al. (2007) High-throughput analysis of promoter occupancy reveals direct neural targets of FOXP2, a gene mutated in speech and language disorders. *Am J Hum Genet* 81:1232–50. Available at: http://www.pubmedcentral.nih.gov/articlerender.fcgi?artid=2276341&tool=pmcentrez&rendertype=abstract [Accessed June 3, 2014].

44. Spiteri E et al. (2007) Identification of the transcriptional targets of FOXP2, a gene linked to speech and language, in developing human brain. *Am J Hum Genet* 81:1144–57. Available at: http://www.pubmedcentral.nih.gov/articlerender.fcgi?artid=2276350&tool=pmcentrez&rendertype=abstract [Accessed June 27, 2014].

45. Rouault TA (2013) Iron metabolism in the CNS: implications for neurodegenerative diseases. *Nat Rev Neurosci* 14:551–64. Available at: http://www.ncbi.nlm.nih.gov/pubmed/23820773 [Accessed May 27, 2014].

46. Kadish I et al. (2009) Hippocampal and cognitive aging across the lifespan: a bioenergetic shift precedes and increased cholesterol trafficking parallels memory impairment. *J Neurosci* 29:1805–16. Available at: http://www.pubmedcentral.nih.gov/articlerender.fcgi?artid=2661568&tool=pmcentrez&rendertype=abstract [Accessed June 18, 2014].

47. Collum RG, Clayton DF, Alt FW (1991) Structure and expression of canary myc family genes. *Mol Cell Biol* 11:1770–6. Available at: http://www.pubmedcentral.nih.gov/articlerender.fcgi?artid=369496&tool=pmcentrez&rendertype=abstract [Accessed July 8, 2014].

48. Kurosawa Y et al. (2012) Cyclocreatine treatment improves cognition in mice with creatine transporter deficiency. *J Clin Invest* 122:2837–46. Available at: http://www.pubmedcentral.nih.gov/articlerender.fcgi?artid=3408730&tool=pmcentrez&rendertype=abstract [Accessed July 8, 2014].

49. Puusepp H et al. (2010) The screening of SLC6A8 deficiency among Estonian families with X-linked mental retardation. *J Inherit Metab Dis* 33 Suppl 3:S5–11. Available at: http://www.ncbi.nlm.nih.gov/pubmed/24137762 [Accessed July 8, 2014].

50. Rosenberg EH et al. (2004) High prevalence of SLC6A8 deficiency in X-linked mental retardation. *Am J Hum Genet* 75:97–105. Available at: http://www.pubmedcentral.nih.gov/articlerender.fcgi?artid=1182013&tool=pmcentrez&rendertype=abstract [Accessed July 8, 2014].

51. Banerjee S, Riordan M, Bhat MA (2014) Genetic aspects of autism spectrum disorders: insights from animal models. *Front Cell Neurosci* 8:58. Available at: http://www.pubmedcentral.nih.gov/articlerender.fcgi?artid=3932417&tool=pmcentrez&rendertype=abstract [Accessed June 2, 2014].

52. Schwarzbraun T et al. (2004) Cloning, genomic structure, and expression profiles of TULIP1 (GARNL1), a brain-expressed candidate gene for 14q13-linked neurological phenotypes, and its murine homologue. *Genomics* 84:577–86. Available at: http://www.ncbi.nlm.nih.gov/pubmed/15498464 [Accessed July 8, 2014].

53. Shimojima K et al. (2009) TULIP1 (RALGAPA1) haploinsufficiency with brain development delay. *Genomics* 94:414–22. Available at: http://www.ncbi.nlm.nih.gov/pubmed/19733229 [Accessed July 8, 2014].

54. Kerner B et al. (2013) Rare Genomic Variants Link Bipolar Disorder with Anxiety Disorders to CREB-Regulated Intracellular Signaling Pathways. *Front psychiatry* 4:154. Available at: http://www.pubmedcentral.nih.gov/articlerender.fcgi?artid=3842585&tool=pmcentrez&rendertype=abstract [Accessed June 5, 2014].

55. Wolf SS, Patchev VK, Obendorf M (2007) A novel variant of the putative demethylase gene, s-JMJD1C, is a coactivator of the AR. *Arch Biochem Biophys* 460:56–66. Available at: http://www.ncbi.nlm.nih.gov/pubmed/17353003 [Accessed June 11, 2014].

56. Dong E, Gavin DP, Chen Y, Davis J (2012) Upregulation of TET1 and downregulation of APOBEC3A and APOBEC3C in the parietal cortex of psychotic patients. *Transl Psychiatry* 2:e159. Available at: http://www.pubmedcentral.nih.gov/articlerender.fcgi?artid=3565208&tool=pmcentrez&rendertype=abstract [Accessed July 8, 2014].

57. Peters MM, Hill KE, Burk RF, Weeber EJ (2006) Altered hippocampus synaptic function in selenoprotein P deficient mice. *Mol Neurodegener* 1:12. Available at: http://www.pubmedcentral.nih.gov/articlerender.fcgi?artid=1594565&tool=pmcentrez&rendertype=abstract [Accessed July 8, 2014].

58. Pitts MW et al. (2012) Deletion of selenoprotein P results in impaired function of parvalbumin interneurons and alterations in fear learning and sensorimotor gating. *Neuroscience* 208:58–68. Available at: http://www.pubmedcentral.nih.gov/articlerender.fcgi?artid=3362796&tool=pmcentrez&rendertype=abstract [Accessed July 8, 2014].

59. Lee KY, Ahn YM, Joo E-J, Chang JS, Kim YS (2006) The association of DUSP6 gene with schizophrenia and bipolar disorder: its possible role in the development of bipolar disorder. *Mol Psychiatry* 11:425–6. Available at: http://www.ncbi.nlm.nih.gov/pubmed/16491131 [Accessed July 8, 2014].

60. Locatelli D et al. (2012) Human axonal survival of motor neuron (a-SMN) protein stimulates axon growth, cell motility, C-C motif ligand 2 (CCL2), and insulin-like growth factor-1 (IGF1) production. *J Biol Chem* 287:25782–94. Available at: http://www.pubmedcentral.nih.gov/articlerender.fcgi?artid=3406665&tool=pmcentrez&rendertype=abstract [Accessed July 8, 2014].

61. Akten B et al. (2011) Interaction of survival of motor neuron (SMN) and HuD proteins with mRNA cpg15 rescues motor neuron axonal deficits. *Proc Natl Acad Sci U S A* 108:10337–42. Available at: http://www.pubmedcentral.nih.gov/articlerender.fcgi?artid=3121858&tool=pmcentrez&rendertype=abstract [Accessed July 2, 2014].

62. Fu Y et al. NEDD9 gene polymorphism influences the risk of Alzheimer disease and cognitive function in Chinese older persons. *Alzheimer Dis Assoc Disord* 26:88–90. Available at: http://www.ncbi.nlm.nih.gov/pubmed/21399483 [Accessed July 8, 2014].

63. Kiryushko D et al. (2006) Molecular mechanisms of Ca(2+) signaling in neurons induced by the S100A4 protein. *Mol Cell Biol* 26:3625–38. Available at: http://www.pubmedcentral.nih.gov/articlerender.fcgi?artid=1447425&tool=pmcentrez&rendertype=abstract [Accessed July 8, 2014].
